# miR-31 affects colorectal cancer cells by inhibiting autophagy in cancer-associated fibroblasts

**DOI:** 10.18632/oncotarget.12873

**Published:** 2016-10-25

**Authors:** Xiaodong Yang, Xiaohui Xu, Junjia Zhu, Shuyu Zhang, Yong Wu, Yongyou Wu, Kui Zhao, Chungen Xing, Jianping Cao, Hong Zhu, Ming Li, Zhenyu Ye, Wei Peng

**Affiliations:** ^1^ Department of General Surgery, The Second Affiliated Hospital of Soochow University, Suzhou 215004, China; ^2^ Department of General Surgery, The First People's Hospital of Taicang City, Taicang Affiliated Hospital of Soochow University, Suzhou 215400, China; ^3^ School of Radiation Medicine and Protection, Medical College of Soochow University, Suzhou 215123, China; ^4^ Oncology Department, The First Affiliated Hospital of Soochow University, Suzhou 215006, China

**Keywords:** miR-31, colorectal cancer, CAFs, autophagy, biological behaviors

## Abstract

Autophagy is a double-edged sword in tumor development. Recent studies have found that miRNAs have an inhibitory effect on the regulation of autophagy. It has been reported that miR-31 plays an important role in the development of colorectal cancer. However, what role miR-31 plays in colorectal cancer-associated fibroblasts (CAFs) has not been determined. In this study, we confirmed that the expression of miR-31 in CAFs was higher than in normal colorectal fibroblasts (NFs). We also found that treatment of CAFs with miR-31 mimic inhibited the expression of the autophagy-related genes Beclin-1, ATG, DRAM and LC3. In addition, we found up-regulation of miR-31 significantly affected colorectal cancer cell behaviors, including proliferation, invasion and apoptosis. Also, up-regulation of miR-31 in CAF could increase the radiosensitivity of colorectal cancer cells co-cultured with CAF. In summary, miR-31 can inhibit autophagy in colorectal CAFs, affect colorectal cancer development, and increase the radiosensitivity of colorectal cancer cells co-cultured with CAF. We hypothesize that miR-31 may become a new target of treatments for colorectal cancer.

## INTRODUCTION

The tumor microenvironment is a tumor pathology-related environment that consists of tumor cells, stromal cells, cytokines, immune cells and other components. This environment is characterized by tissue hypoxia, acidosis, and interstitial pressure. A large number of growth factors, proteolytic enzymes, and immune inflammatory response factors in the tumor microenvironment act jointly on the surface of tumor cells and have an important impact on cell proliferation, metastasis, and differentiation. Cancer-associated fibroblasts (CAFs), as the major type of tumor stromal cells, display obvious differences in biological characteristics that make them distinct from normal tissue stromal fibroblasts (NFs). CAFs not only provide nutritional support for the passive growth of the tumor, they also promote the evolution of the tumor by regulating metabolism, immune responses, and the epithelial-mesenchymal transition. In addition, a variety of CAF-secreted active factors interact with epithelial cells, endothelial cells, pericytes and inflammatory cells to promote tumor evolution by regulating metabolism and immune responses. As a result of the above interactions, CAFs are becoming a new target for anti-cancer therapies. [1]

Pavlides et al. [2] proposed a “tumor metabolism autophagic interstitial model” that was called “the reverse Warburg effect” in 2009. The authors found that the autophagy of CAFs might be involved in tumor energy acquisition, which might provide energy for tumor cell survival, proliferation, invasion and metastasis [3, 4]. By co-culturing CAFs and tumor cells, the authors confirmed that breast cancer cells, prostate cancer cells and other tumor cells retained strong mitochondrial function and aerobic oxidation. CAFs not only induced tumor cell mitochondrial formation and suppressed tumor cell apoptosis, but CAF autophagy also directly affected the ability of tumor cells to proliferate, invade and metastasize by altering energy metabolic pathways. In addition, studies have shown that the level of autophagy observed in CAFs changes the biological characteristics of the tumor cells and thereby affects their responses to different treatments [5–7]. Therefore, CAFs, as a treatment target, are likely to create new therapeutic options for tumors.

Micro-RNAs (miRNAs) are a group of small non-coding RNAs that are approximately 22 (18 to 25) nucleotides (nt) in length. miRNAs have been found to be associated with a variety of diseases, including cancers. A growing number of studies have confirmed that miRNAs play essential roles in the development, diagnosis, treatment and prognosis of a variety of tumors. There is an increasing trend toward using miRNAs as biomarkers for the diagnosis of cancers and as target molecules for cancer treatment [8].

miR-31 is a type of common miRNA, and its target genes are involved in the regulation of autophagy, according to a bioinformatic prediction of its target genes. miR-31 plays an important role in the development of colorectal cancer. Overexpression of miR-31 promotes the incidence of colorectal cancer [9–11], while down-regulation of miR-31, miR-9 and miR-182 promotes the proliferation and survival of colorectal cancer [12]. miRNA-31 contributes to colorectal cancer development by targeting factorinhibiting HIF-1α (FIH-1) [13], and miR-31 is closely associated with the colorectal cancer stage [14]. In addition, miR-31 enhances the sensitivity of colorectal cancer cells to fluorouracil (5-FU) and promotes their invasion and metastasis [15–17]. However, few studies have investigated the relationship between miR-31 and CAFs. Olga Aprelikova found that miR-31 is the most down-regulated miRNA in endometrial CAFs and that overexpression of miR-31 significantly impairs the ability of CAFs to stimulate tumor cell migration and invasion [18]. Mitra [19] found that miR-31 and miR-214 were down-regulated in ovarian CAFs and that miR-155 was up-regulated compared with NFs or tumor-adjacent fibroblasts; Mimicking this deregulation by transfecting miRNAs and miRNA inhibitors induces the functional conversion of NFs into CAFs, and the reverse experiment resulted in the reversion of CAFs into NFs.

In summary, miR-31 is involved in the phenotypic conversion of CAFs and plays an important role in regulating the biological functions of CAFs. We therefore hypothesize that miR-31 may be involved in the regulation of autophagy in CAFs and may thereby affect the biological properties of colorectal cancer.

## RESULTS

### Isolation and identification of colorectal cancer-associated fibroblasts and normal fibroblasts

**Figure 1:**
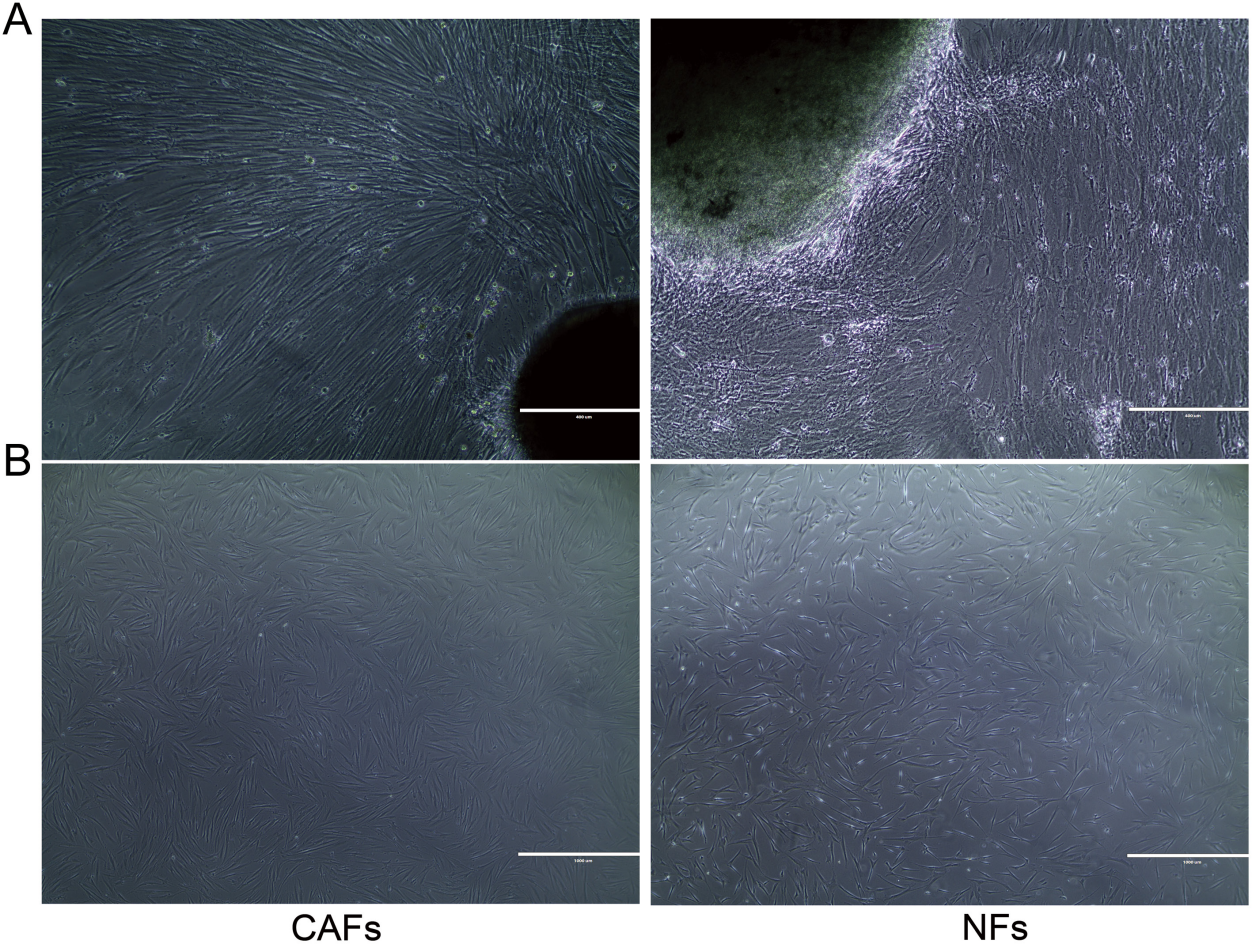
Primary culture methods were used to cultivate CAFs and NFs **A.** Approximately 10 days after primary culture, a large number of CAFs/NFs emerged from the tissues. **B.** CAFs/NFs were stably passaged after three generations. Primary culture is an effective method for cultivating CAFs/NFs.

**Figure 2:**
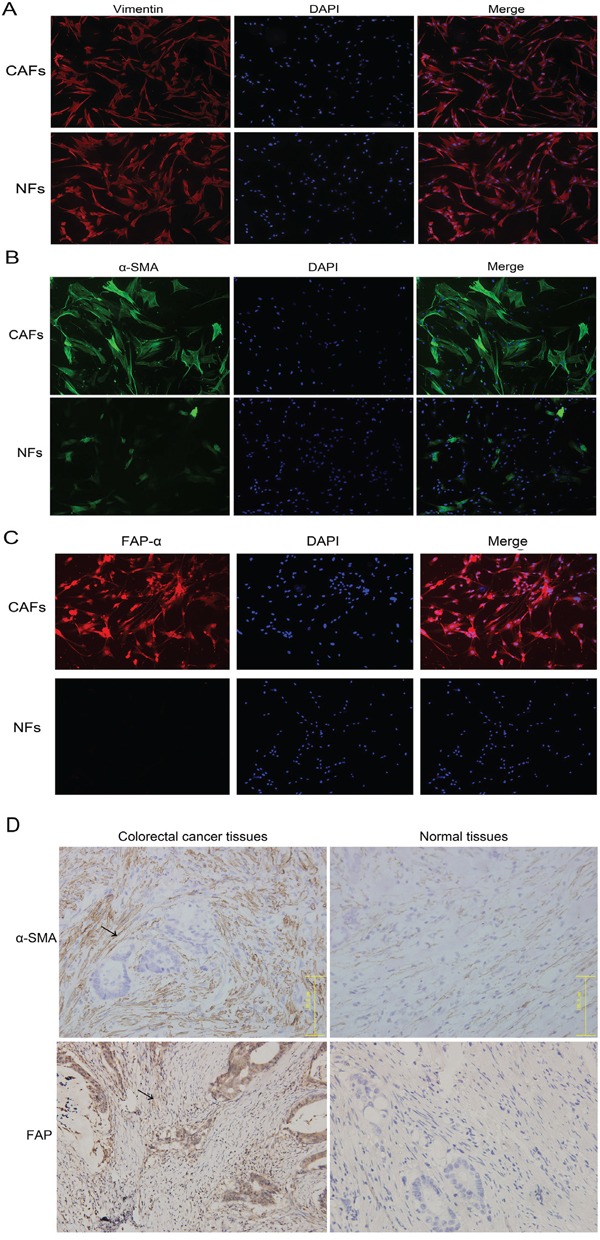
CAF and NF distinction and identification and immunohistochemistry was used to locate the positions of CAFs/NFs in colorectal cancer and normal tissues **A.** Vimentin was expressed in both CAFs and NFs. **B.** α-SMA was highly expressed in CAFs but was expressed at lower levels in NFs. **C.** FAP-α was only expressed in CAFs. α-SMA and FAP-α can be used as positive markers for CAFs. **D.** α-SMA was highly expressed in tumor interstitial tissue and showed lower expression in normal tissues. FAP-α was highly expressed in tumor tissues and rarely expressed in normal tissues.

Primary culture methods were used to cultivate CAFs and NFs (Figure 1). Immunofluorescence was used to detect related proteins, including α-SMA, FAP-α and Vimentin, to screen the CAFs and NFs. α-SMA and FAP-α were highly expressed in CAFs but were expressed at lower levels in NFs, and there were significant differences between them (Figure 2B, 2C). Vimentin was expressed in both types of cells, and no differences were found (Figure 2A).

Immunohistochemistry was used to locate CAFs/NFs in colorectal cancer and normal tissues (Figure 2D).

### miR-31 expression differs significantly between CAFs and NFs and up- and down-regulation of miR-31 has no effect on CAF phenotypes, proliferation and apoptosis

**Figure 3:**
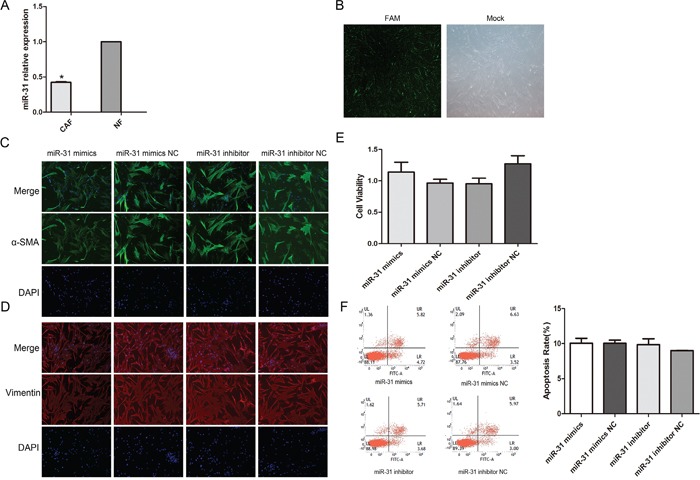
The expression level of miR-31 was significantly lower in CAFs than in NFs and Up- or down-regulation of miR-31 had no effect on the CAF phenotype **A.** The expression of miR-31 in CAF was significantly lower than in NF (*P < 0.05). **B.** transfection efficiency of CAF were transfected with FAM-siRNA, GFP-green fluorescence was detected with a fluorescence microscopy after 48 hours. **C.** No significant difference was observed in the expression of the marker protein α-SMA after the up- or down-regulation of miR-31. **D.** No significant difference was observed in the expression of the marker protein Vimentin after the up- or down-regulation of miR-31. miR-31 had no effect on the CAF phenotype. **E.** No significant difference was observed in the proliferation of CAF after the up- or down-regulation of miR-31. **F.** No significant difference was observed in the apoptosis of CAF after the up- or down-regulation of miR-31.

Real-time quantitative PCR was used to detect the expression of miR-31 in CAFs and matched NFs in three patients. We found that miR-31 expression in CAFs was significantly lower than in NFs (Figure 3A). Real-time quantitative PCR was also used to verify the efficiency of miR-31 transfection (The above results are not given). To assess the transfection efficiency, CAFs were transfected with FAM-siRNA and the transfection efficiency was estimated about 90% (Figure 3B).

miR-31 mimics or an inhibitor were transfected to up- or down-regulate miR-31 expression, respectively. At 48 h after transfection, immunofluorescence was used to detect the effect of miR-31 expression of the surface proteins α-SMA and Vimentin; MTT was used to detect the proliferation of CAF and Flow cytometry analysis was used to detect the apoptosis of CAF. The results showed that the up- or down-regulation of miR-31 had no effect on the CAF phenotype, proliferation and apoptosis (Figure 3C, 3D, 3E, 3F).

### Up- and down-regulation of miR-31 expression affects the CAF autophagy activation

**Figure 4:**
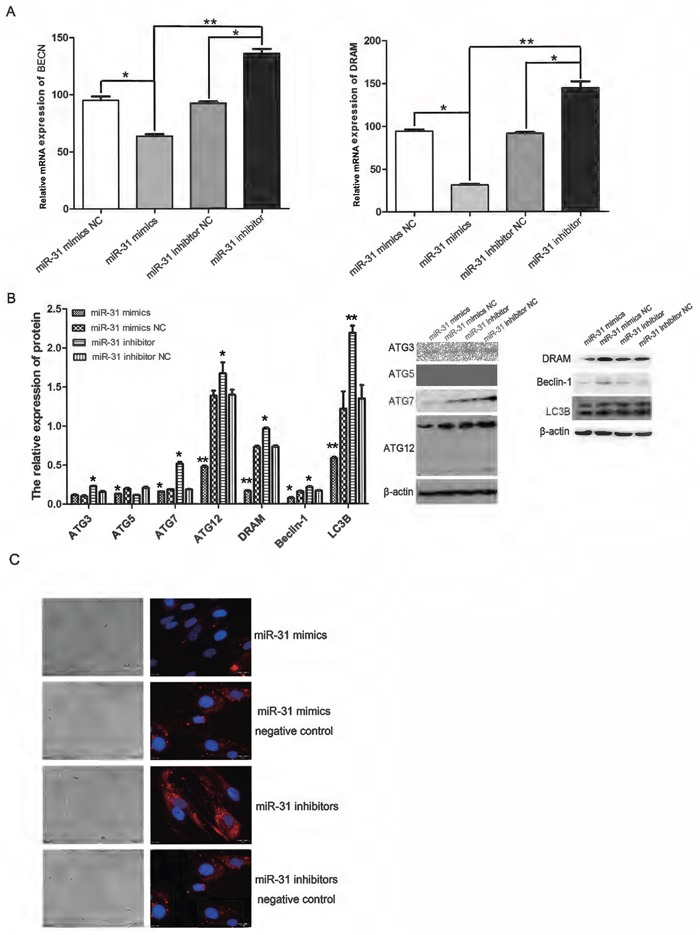
Up- or down-regulation of miR-31 in CAFs inhibited and promoted, respectively, the expression of autophagy-related genes at both the protein and RNA level **A.** At the RNA level, the up-regulation of miR-31 significantly inhibited BECN and DRAM mRNA expression (*P < 0.05), whereas the down-regulation of miR-31 significantly promoted the expression of the above two mRNAs (*P < 0.05), and the differences were significant (**P < 0.01). **B.** At the protein level, the up-regulation of miR-31 significantly inhibited ATG5, ATG7, ATG12, Beclin-1, DRAM and LC3B expression (*P < 0.05), whereas the down-regulation of miR-31 significantly promoted ATG3, ATG7, ATG12, Beclin-1, DRAM and LC3B expression (*P < 0.05), and the differences were significant (**P < 0.01). **C.** Immunofluorescence showed that LC3 expression was significantly reduced after up-regulation of miR-31, and LC3 expression was increased after down-regulation of miR-31.

Real-time quantitative PCR was used to detect the expression of BECN and DRAM RNA, and we found that both were reduced by the up-regulation of miR-31, and vice versa (Figure 4A).

Immunofluorescence and western blot analysis were used to detect the expression of the autophagy-related proteins Beclin-1, ATG, DRAM and LC3. The results showed that miR-31 up-regulation inhibited the expression of the autophagy-related proteins Beclin-1, ATG, DRAM and LC3, while the down-regulation of miR-31 promoted their expression (Figure 4B, 4C).

To further confirm miR-31 affect the autophagy activity of CAF, electron microscopy was used to detect the formation of autophagosomes. Results showed that compared with control and ATG5 shRNA the number of autophagosomes and lysosomes decreased significantly after miR-31 mimics. However, both of them increased after treated with miR-31 inhibitor.

**Figure 5:**
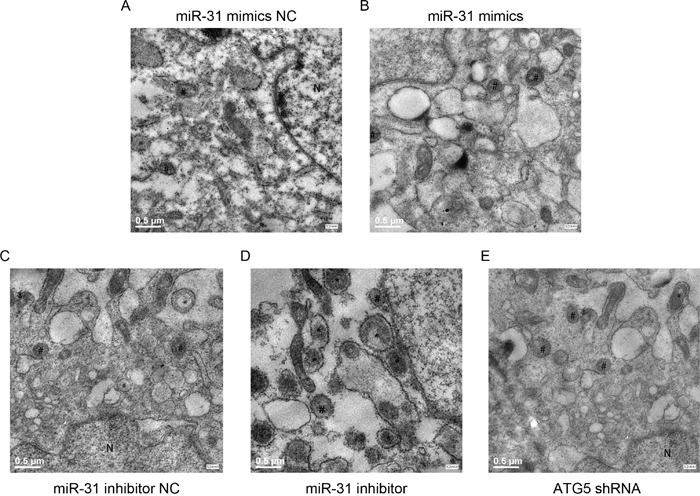
Up- or down-regulation of miR-31 in CAFs inhibited and promoted the formation of the autophagosome **A.** The number and morphology of autophagy and lysosomal in CAF were normal. **B.** There is almost no autophagosomes in CAF after transfected with miR-31 mimics. **C.** The number and morphology of autophagy and lysosomal in CAF were normal. **D.** the number of autophagosomes increased and the morphology is big in CAF after transfected with miR-31 inhibitor. **E.** Autophagosomes were less and small in CAF after transfected with ATG5 shRNA. Scale bars: 0.5 μm. *, autophagosomes; #, lysosomes .Scale bars: 0.5 μm. *, autophagosomes; #, lysosomes.

These results suggest MiR-31 can significantly inhibit the activation of autophagy in CAF (Figure 5).

### Up- and down-regulation of miR-31 in CAFs affects the migration and radiosensitivity of the co-cultured colorectal cancer cell lines HCT116 and HCT-8

**Figure 6:**
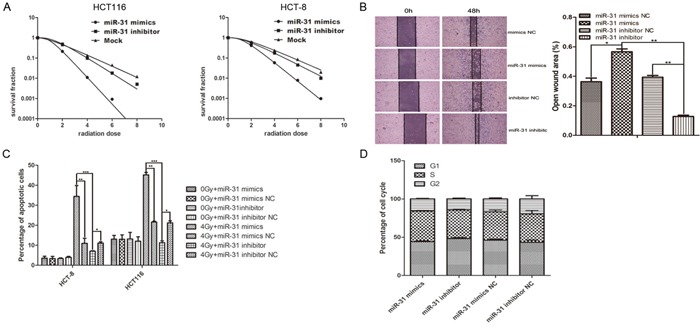
Up- or down-regulation of miR-31 in CAFs regulated the radiosensitivity and X-ray-induced apoptosis of co-cultured CRC cells **A.** (Left) HCT116 cells were exposed to X-rays at different irradiation doses, 0, 2, 4, 6, and 8 Gy, after co-culture with CAFs that were transfected with miR-31 mimics, miR-31 inhibitor or mock (negative control) for 48 h. After 14 days, the surviving fraction was calculated as a ratio of the number of colonies formed divided by the total number of cells plated times the plating efficiency. (Right) the surviving fraction of HCT-8 In all, up- and down-regulation of miR-31 in CAFs affected the radiation sensitivity of colorectal cancer cells. **B.** Wound healing assay of HCT-8 cells co-cultured with CAF regulated miR-31. Wound healing was observed 48 h after the treatment, and the open wound area was normalized to the area at the initial time that the wound was made. The data are presented as the means ± SEM and normalized to the control cells (*P < 0.05, **P < 0.01) **C.** Flow cytometry assays were used to determine the amount of apoptosis that occurred in HCT116 and HCT-8 cells that were co-cultured with CAFs transfected with miR-31 mimic, a miR-31 mimic negative control, miR-31 inhibitor or a miR-31 inhibitor negative control. The percentage of apoptotic cells was determined in the non-transfected and transfected cells were that subjected to 0 and 8-Gy irradiation. The data are shown as the mean ± SEM of three independent experiments. The miR-31 mimic significantly increased X-ray-induced apoptosis in both cell lines compared with the miR-31 mimic negative control (**P < 0.01) and the miR-31 inhibitor (***P < 0.001), and the miR-31 inhibitor decreased X-ray-induced apoptosis in both cell lines compared with the miR-31 inhibitor negative control (*P < 0.05). **D.** miR-31 regulated in CAF had no effect on cell cycle progression of colorectal cancer cells HCT-8. Cell cycle analyses were done on co-cultured HCT-8, cells transduced with PI in preparation for flow cytometry with the FACScan/Cell FIT system as described in Materials and methods. Wound healing assay of HCT-8 cells co-cultured with CAF regulated miR-31. Wound healing was observed 48 h after the treatment, and the open wound area was normalized to the area at the initial time that the wound was made. The data are presented as the means ± SEM and normalized to the control cells.

Clonogenic assays were used to determine how miR-31 in CAFs affected the radiosensitivity of colorectal cancer cells that were co-cultured, and we observed a high clonal efficiency in the miR-31-down-regulated group of CAFs in both the HCT8 and HCT116 cell lines (Figure 6A). The cell migration rates of HCT-8 co-cultured with CAF transfected with miR-31 mimics was significantly suppressed; on the contrary, CAF transfected with miR-31 inhibitor could significantly increase the cell migration rates of HCT-8 that co-cultured (Figure 6B). Flow cytometry and cell cycle analysis showed that miR-31 up-regulation significantly promoted apoptosis in CRC cells but had no effect on cell cycle (Figure 6C, 6D).

## DISCUSSION

miRNAs in CAFs play an important role in the biological behavior of tumors. Our study suggests that miR-31 can significantly affect the autophagy of colorectal cancer CAF in both protein and mRNA levels and further affect the proliferation and radiosensitivity (mainly radiation-induced apoptosis) of colorectal cancer cells.

In recent years, many studies have found that miRNAs are associated with tumor cell autophagy. However, the identified miRNAs participate in the negative regulation of tumor cell autophagy pathways. miRNAs can be divided into carcinogenic and tumor suppressor miRNAs that act during tumor formation. Carcinogenic miRNAs include miR-106a and miR-183; miR-106a prevents cell autophagy by blocking the activation of the autophagy induction complex ULK1, while miR-183 hinders LC3B1 activation and LC3-II activity and negatively regulates autophagy vesicle elongation and conformation. The tumor suppressor miRNAs include miR-30a and miR-101; miRNA-30a inhibits the activity of the autophagy gene Beclin-1 to prevent autophagic vesicle nucleation, while miR-101 suppresses the regulation of endocytosis by RAB5A and thereby affects autophagy body formation during the early stages of autophagosome formation [20]. Studies have also reported that miRNAs play regulatory roles in the autophagy of colon cancer cells. For example, Qased AB found that overexpression of miR-18a inhibited mTORC1 activity to prevent the occurrence of colon cancer in HCT116 cells through the induction of autophagy [21]. Therefore, miRNAs regulate all stages of tumor cell autophagy.

There are also reports that show that miRNAs are differentially expressed in CAFs and NFs. miRNAs are not only involved in the phenotypic conversion between CAF and NF, but they also play an important role in the regulation of tumor-promoting functions in CAFs. Enkelmann [22] found that there are proteomic and epigenetic differences between urinary bladder carcinoma NFs and CAFs and identified the specific protein expression patterns and miRNA profiles of CAFs compared with NFs. Similarly, Liuyang Zhao explored the difference between CAFs and NFs in breast cancer and surmised that differentially expressed miRNAs might be involved in the tumor-promoting function of CAFs in breast cancer [23]. However, no report has shown whether miRNAs regulate autophagy in CAFs.

miR-31 is a potential tumor-related miRNA that has been shown to be up-regulated in a variety of human cancers as well as colorectal cancer. Lei et al. [17] found that miR-31 was up-regulated in colorectal cancer and that the inhibition of miR-31 reduced cell growth and invasion, indicating that it functioned as an oncogene. In addition, a direct target gene, CDKN2B, was identified for miR-31, and the results indicated that miR-31 may act by suppressing CDKN2B. Therefore, miR-31 could be used as a novel biomarker for the diagnosis and prognosis of colorectal cancer.

Similarly, Nosho et al. [24] found that high levels of miR-31 expression were associated with BRAF mutations in a large number of colorectal cancers. Their study indicated that an antitumor effect was observed as a result of the transfection of a miR-31 inhibitor. Thus, miR-31 may be a promising diagnostic biomarker and therapeutic target for patients with colorectal cancer. Otherwise, in colorectal cancer, high miR-31 expression has been correlated with an advanced stage of the disease [25].

Therefore, miR-31 has been shown to affect the development, staging, and prognosis of colorectal cancer and it is expected that it will become a major target for the diagnosis and prognosis of colorectal cancer.

miR-31 in CAFs affects the occurrence and development of certain cancers. For example, the down-regulation of miR-31 in CAFs and the corresponding increased expression of the homeobox gene SATB2 contributed to the migration and invasiveness of endometrial tumor cells [18].

miR-31, acting as an oncogenic gene, has been reported to be involved in modulating cellular processes that lead to tumor occurrence, development, and drug resistance. miR-31 also regulates the expression of several target-suppressive or oncogenic genes, such as ARID1A, SATB2, EMSY, and ABCB9, to affect tumorigenesis and tumor progression and prognosis [26]. However, the mechanism by which miR-31 in colorectal CAFs regulates the biological behavior of colorectal cancer has not been reported. The starting point of this study was to determine how miR-31 regulates CAF autophagy to thereby affect colorectal cancer. We found that the inhibition of miR-31 up-regulated autophagy in colorectal CAFs and promoted colorectal cancer cell proliferation, invasion, and metastasis in a co-culture system in addition to increasing the radiosensitivity of colorectal cancer cells. Although we know that miR-31 may affect other pathways in CAFs to alter the biological behavior of colorectal cancer, the idea that the autophagy of CAFs provides energy to the tumor via the “reverse Warburg effect” [2, 27] is currently an important viewpoint. We therefore focused on determining whether and how miR-31 might regulate autophagy in CAFs throughout this study.

In summary, in the present study, we demonstrate that miR-31 can regulate the autophagy of CAFs and thereby regulate the proliferation, metastasis, and radiosensitivity of colorectal cancer cells. There are limitations to the current study. The results mostly describe the phenomena, and studies of related mechanisms and *in vivo* experimental studies were not involved. We will therefore continue this research and hope that the results of *in vivo* experiments are consistent with the results of the current study. In addition, through a study of mechanisms, we hope to identify the regulatory targets of miR-31 in CAF autophagy. Based on the above data, we show that miR-31 is an important target for the treatment of colorectal cancer.

## MATERIALS AND METHODS

### Patients

Five colorectal cancer patients (approximately 60 years of age) who were undergoing colorectomy without preoperative radiotherapy and chemotherapy were selected. Fresh colorectal cancer tissues and normal colorectal tissues were obtained from these patients. Non-tumorous tissues were harvested from the intestinal mucosa 5 cm from the tumor site, whereas normal control tissues were harvested from the intestinal mucosa over 10 cm from the tumor site.

### Clinical samples

Three colorectal cancer specimens were collected after first resection of the primary lesions from patients at the Second Affiliated Hospital of Soochow University. For the control samples, fresh colon tumor tissue specimens were collected at the same pathological stage from an area 8 cm from the border of the tumor tissues. This study was conducted in accordance with the Declaration of Helsinki and with approval from the Ethics Committee of Soochow University. Written informed consent was obtained from all participants. No patients had received any treatment prior to surgery.

### Cell lines

Paired samples (normal and tumor) from each patient were placed in RPMI-1640 medium (HyClone, Logan, UT, USA) containing 4% meropenem (Haibin Pharmaceutical Co., Ltd, Shenzhen, China). Tissues were cut with a scalpel into pieces of approximately 3 mm^3^, placed in 25-cm^2^ culture flasks and covered with 1 ml of fetal bovine serum (FBS; HyClone, Logan, UT, USA). The flasks were then inverted for 48 h. After 48 h, RPMI-1640 medium supplemented with 20% FBS (HyClone, Logan, UT, USA) was added to the flasks, and the flasks were turned over. Cultures were incubated in humidified air containing 5% CO_2_ at 37°C. Following tissue attachment (2–3 days), the culture medium was changed twice per week for the next 2–3 weeks. Under these conditions, fibroblasts were explanted from tissue fragments, while other cells were mostly retained within the tissue. Fibroblasts formed multiple dense colonies that spread out on the culture dish. After 10-14 days, the cultured cells were briefly trypsinized and replated into flasks (passage 1). After reaching confluence, the cultured cells were split 1:2 every 3–4 days. All fibroblasts used in experiments were between passages 4 and 9 [18].

### Immunofluorescence

Fibroblasts, including CAFs and NFs, at passage 3 that were treated with miR-31 mimics, a mimic negative control, a miR-31 inhibitor or inhibitor negative controls were grown on coverslips in 24-well plates, fixed with 4% paraformaldehyde for 30 min, permeabilized with 0.1% Triton X-100 for 10 min and blocked with 1% bovine serum albumin (BSA) for 1 h. Then, the cells were incubated with primary antibodies against anti-stromal cell-derived factor 1 (SDF-1) (Epitomics, Burlingame, California, United States, dilution 1:100) or anti-alpha-smooth muscle actin (α-SMA) and LC3 (Abcam, dilution 1:100) at 4°C overnight. After the cells were washed with phosphate-buffered saline (PBS), the samples were stained with a fluorescein isothiocyanate (FITC)-labeled goat anti-rabbit secondary antibody (1:1000) for 1 h at 37°C in a dark room and then labeled with DAPI (4',6-diamidino-2-phenylindole) for 5 minutes. The coverslips were blocked with Antifade Mounting Medium. Immunofluorescent images were captured using a ZEISS Observer.A1 microscope (Observer.A1, Germany).

### Cell proliferation assay

The cells were seeded in a 96-well plate at a density of 2 × 10^^3^ cells per well. CAFs were transfected with 50 nM of either miR-31 mimics and miR31mimics NC or miR-31 inhibitor and miR-31 inhibitor NC 24 h later and allowed to grow for another 48 h. The cell proliferation was measured using the 3-(4,5-dimethylthiazol-2-yl)-2,5-diphenyl-2Htetrazolium bromide (MTT) assay 48 h after the transfection with the RNAs. Briefly, 20 μL of the MTT solution (5 mg/mL) was added to each well, and the cells were incubated for another 4 h at 37°C. The medium was then aspirated, and 150 μL of dimethylsulfoxide (DMSO) was added to dissolve the crystals. The optical density was measured at 492 nm using a microplate reader (Bio-Rad, Hercules, CA, USA). The viability index was calculated as the experimental OD value/the control OD value. Three independent experiments were performed in quadruplicate.

### miRNA target prediction

The analysis used to predict miR-31 targets was performed using algorithms from the miRecords (http://mirecords.umn.edu/miRecords/), TargetScan (http://targetscan.org/), PicTar (http://pictar.mdc-berlin.de/), and miRanda (http://www.microrna.org/microrna/home.do) website tools.

### Quantitative real-time RT-PCR

Total RNA was extracted from the oncocytes in the sections using the TRIzol one-step method according to the manufacturer's instructions (Invitrogen, Carlsbad, CA, USA). Total RNA was collected according to the manufacturer's instructions with a Recover All Total Nucleic Acid Isolation kit using TRIzol reagent (Applied Biosystems, Carlsbad, CA, USA). Light absorption values were read at 230, 260 and 280 nm using a spectrophotometer to determine purity and density. Then, qRT-PCR was performed according to the kit manufacturer's instructions (Hairpin-it^TM^ miRNAs qPCR Quantification Kit) and the instrument manufacturer's instructions (CFX96™And CFX38 4™ Real time PCR detection system, Bio-Rad, USA). Gene amplification was detected using SYBR dye, and the U6 gene was used to normalize each sample. qRT-PCR was also used to confirm the up-regulation of miR-31 expression after transfection and the expression of Beclin-1 and DRAM mRNA. miR-31 was amplified using an reverse transcription (RT) primer (5′-GTCGTATCCAGTGCAGGGTCCGAGGTATTCGCACTGGATA CGACAGCTAT-3′), a reverse primer (5′-AGGCAAGAT GCTGGCATAGCT-3′) and a forward primer (5′-GCGGCGGAGGCAAGATGCTGGC-3′).

To assess the transfection efficiency, CAFs were transfected with FAM-siRNA (sense: 5′-UUCUCCGA ACGUGUCACGUTT-3′; antisense: 5′-ACGUGACAC GUUCGGAGAATT-3′) 48 hours after transfection, the expression of green fluorescent protein (GFP) was detected with a fluorescence microscopy. The transfection efficiency was estimated about 90%.

To inhibit autophagy as control in, ATG5 siRNAs (GCAACTCTGGATGGGATTG, ATG5 siRNA1; CATCTGAGCTACCCGGATA, ATG5 siRNA2) were also synthesized.

### Immunohistochemical analysis

The paraffin sections were dewaxed to water. Tissues were incubated in 3% H_2_O_2_ for 10 min at room temperature to remove endogenous peroxidase activity and then rinsed in distilled water then PBS for 5 min each. The tissue was blocked in 10% BSA, sealed, and incubated at room temperature for 10 min. The tissue was then incubated with anti-α-SMA or fibroblast activation protein (FAP; 1:500) antibodies at 37°C. Tissues were washed 3 times in PBS for 5 min each, incubated with secondary antibody (1: 1000 dilution) at 37°C for 30 min, and again washed 3 time in PBS for 5 min each. The tissues were then incubated in a solution containing an appropriate dilution of horseradish peroxidase-labeled streptavidin (diluted in PBS) at 37°C for 30 min and then washed 3 times in PBS for 5 min each. The tissue was then incubated with DAB chromogenic reagent. The tissues were rinsed thoroughly with water, stained, and mounted before being imaged using a microscope camera.

### Wound healing migration assays

Cells were co-cultureed with CAF that up/down-regulation of miR-31. After 48h, they were seeded onto 6-well plates and allowed to form a confluent monolayer for 24 h. After treatment, the monolayer was scratched with the tip of a 200 μL pipette and then washed twice with PBS to remove the floating and detached cells. Then, fresh serum-free medium was added, and photos were taken at 0, 48 h to assess cell migration using a microscope (Olympus, Tokyo, Japan).

### Cell apoptosis assays and cell cycle analysis

48h later, following conventional digestion, CAFs that up/down-regulation of miR-31 and cells that were co-cultured with CAFs transfected in the logarithmic growth phase were used to prepare a single cell suspension, 2 × 10^5 cells/ml was seeded in 6-well plates, and two vice-holes were set. The cells were subjected to X-ray irradiation 24 h after adhesion.

After 48 h, all cells were collected and centrifuged at 2,000 rpm for 5 min; the supernatant was discarded after washing with PBS and centrifugation was repeated twice. The cells were then stained with fluorescein FITC-conjugated Annexin V and PI (KeyGen, Nanjing, China) and analyzed by flow cytometry (Beckman Coulter, Brea, CA, USA). For test results, the ratio of early apoptosis cells was only to be counted.

As well as cell apoptosis assays, 48 h after X-ray irradiation, all cells were collected and centrifuged at 1,000 rpm for 5 min; the supernatant was discarded after washing with PBS and centrifugation was repeated twice. Cells were trypsinized and fixed with 70% ethanol/PBS. Cell cycle analysis was carried out by staining DNA with PI in preparation for flow cytometry with the FACScan/Cell FIT system (Becton–Dickinson, San Jose, CA, USA) and quantified with Modfit software.

### Western blot

Fibroblast lysates were prepared after transfection by incubating the cells in lysis buffer (125 mM Tris–HCl, pH 6.8, 2% SDS, 10% v/v glycerol) at 4°C for 30 min. Protein concentrations were determined using bicinchoninic acid (BCA) protein assays (Bio-Rad Laboratories, Hercules, CA, USA). A total of 40 μg of cell lysate was subjected to 12% sodium dodecyl sulfate-polyacrylamide gel electrophoresis (SDS-PAGE). Following SDS-PAGE, the proteins were transferred onto a polyvinylidene fluoride (PVDF) membrane (Millipore, Temecula, CA, USA), which was blocked with 10% dry milk in Tris-buffered saline containing Tween-20 (TBST). The membrane was then probed with primary antibodies at 4°C for 12 h. Primary antibodies directed against Beclin-1 (Cell Signaling Technology, Boston, MA, USA), ATG5, ATG7, and ATG12 (Cell Signaling Technology, Boston, MA, USA), DRAM (Abcam, London, United Kingdom), LC3B (Cell Signaling Technology, CA, USA) and β-actin (Santa Cruz Biotechnology, CA, USA) were used at a dilution of 1:1000.

### Transmission electron microscopy

CAFs were seeded into 6-well plates and transfected with miR-31 mimics, miR31mimics NC, miR-31 inhibitor, miR-31 inhibitor NC and ATG5 shRNA(as autophagy inhibition control) 24 h later and allowed to grow for another 48 h. Cells were then fixed for 2 hours with 2.5% glutaraldehyde in 0.1 M phosphate buffer (pH 7.4), postfixed in 1% OsO4 dehydrated in graded ethanol and then embedded in epoxy resin. Blocks were cut on an ultramicrotome into ultrathin sections, which were poststained with uranyl acetate and lead citrate, and viewed under a Hitachi 7100 electron microscopy.

To inhibit autophagy as control, ATG5 siRNAs (GCAACTCTGGATGGGATTG, ATG5 siRNA1; CATCTGAGCTACCCGGATA, ATG5 siRNA2) were also synthesized.

### Statistical analyses

The data are expressed as the mean ± standard error of the mean (SEM) of at least three independent experiments. Standard error bars are included for all data points. The data were then analyzed using Student's t tests when only two groups were present or assessed by one-way analysis of variance (ANOVA) when more than two groups were compared. Statistical analysis was performed using SPSS software (Release 17.0, SPSS Inc.). The data were considered significant if *P* < 0.05. GraphPad Prism 5 software was used to draw the survival curves.

## References

[1] Castells M, Thibault B, Delord JP, Couderc B (2012). Implication of tumor microenvironment in chemoresistance: tumor-associated stromal cells protect tumor cells from cell death. International journal of molecular sciences.

[2] Pavlides S, Whitaker-Menezes D, Castello-Cros R, Flomenberg N, Witkiewicz AK, Frank PG, Casimiro MC, Wang C, Fortina P, Addya S, Pestell RG, Martinez-Outschoorn UE, Sotgia F, Lisanti MP (2009). The reverse Warburg effect: aerobic glycolysis in cancer associated fibroblasts and the tumor stroma. Cell cycle (Georgetown, Tex).

[3] Bonuccelli G, Whitaker-Menezes D, Castello-Cros R, Pavlides S, Pestell RG, Fatatis A, Witkiewicz AK, Vander Heiden MG, Migneco G, Chiavarina B, Frank PG, Capozza F, Flomenberg N (2010). The reverse Warburg effect: glycolysis inhibitors prevent the tumor promoting effects of caveolin-1 deficient cancer associated fibroblasts. Cell cycle (Georgetown, Tex).

[4] Bonuccelli G, Tsirigos A, Whitaker-Menezes D, Pavlides S, Pestell RG, Chiavarina B, Frank PG, Flomenberg N, Howell A, Martinez-Outschoorn UE, Sotgia F, Lisanti MP (2010). Ketones and lactate “fuel” tumor growth and metastasis: Evidence that epithelial cancer cells use oxidative mitochondrial metabolism. Cell cycle (Georgetown, Tex).

[5] Castello-Cros R, Bonuccelli G, Molchansky A, Capozza F, Witkiewicz AK, Birbe RC, Howell A, Pestell RG, Whitaker-Menezes D, Sotgia F, Lisanti MP (2011). Matrix remodeling stimulates stromal autophagy, “fueling” cancer cell mitochondrial metabolism and metastasis. Cell cycle (Georgetown, Tex).

[6] Capparelli C, Whitaker-Menezes D, Guido C, Balliet R, Pestell TG, Howell A, Sneddon S, Pestell RG, Martinez-Outschoorn U, Lisanti MP, Sotgia F (2012). CTGF drives autophagy, glycolysis and senescence in cancer-associated fibroblasts via HIF1 activation, metabolically promoting tumor growth. Cell cycle (Georgetown, Tex).

[7] Capparelli C, Guido C, Whitaker-Menezes D, Bonuccelli G, Balliet R, Pestell TG, Goldberg AF, Pestell RG, Howell A, Sneddon S, Birbe R, Tsirigos A, Martinez-Outschoorn U, Sotgia F, Lisanti MP (2012). Autophagy and senescence in cancer-associated fibroblasts metabolically supports tumor growth and metastasis via glycolysis and ketone production. Cell cycle (Georgetown, Tex).

[8] Xu X, Yang X, Xing C, Zhang S, Cao J (2013). miRNA: The nemesis of gastric cancer (Review). Oncology letters.

[9] Slaby O, Svoboda M, Fabian P, Smerdova T, Knoflickova D, Bednarikova M, Nenutil R, Vyzula R (2007). Altered expression of miR-21, miR-31, miR-143 and miR-145 is related to clinicopathologic features of colorectal cancer. Oncology.

[10] Wang CJ, Zhou ZG, Wang L, Yang L, Zhou B, Gu J, Chen HY, Sun XF (2009). Clinicopathological significance of microRNA-31, -143 and -145 expression in colorectal cancer. Disease markers.

[11] Xu XM, Qian JC, Deng ZL, Cai Z, Tang T, Wang P, Zhang KH, Cai JP (2012). Expression of miR-21, miR-31, miR-96 and miR-135b is correlated with the clinical parameters of colorectal cancer. Oncology letters.

[12] Cekaite L, Rantala JK, Bruun J, Guriby M, Agesen TH, Danielsen SA, Lind GE, Nesbakken A, Kallioniemi O, Lothe RA, Skotheim RI (2012). MiR-9, -31, and -182 deregulation promote proliferation and tumor cell survival in colon cancer. Neoplasia (New York, NY).

[13] Chen T, Yao LQ, Shi Q, Ren Z, Ye LC, Xu JM, Zhou PH, Zhong YS (2014). MicroRNA-31 contributes to colorectal cancer development by targeting factor inhibiting HIF-1alpha (FIH-1). Cancer biology & therapy.

[14] Chang KH, Miller N, Kheirelseid EA, Lemetre C, Ball GR, Smith MJ, Regan M, McAnena OJ, Kerin MJ (2011). MicroRNA signature analysis in colorectal cancer: identification of expression profiles in stage II tumors associated with aggressive disease. International journal of colorectal disease.

[15] Wang CJ, Stratmann J, Zhou ZG, Sun XF (2010). Suppression of microRNA-31 increases sensitivity to 5-FU at an early stage, and affects cell migration and invasion in HCT-116 colon cancer cells. BMC cancer.

[16] Li Z, Gu X, Fang Y, Xiang J, Chen Z (2012). microRNA expression profiles in human colorectal cancers with brain metastases. Oncology letters.

[17] Lei SL, Zhao H, Yao HL, Chen Y, Lei ZD, Liu KJ, Yang Q (2014). Regulatory roles of microRNA-708 and microRNA-31 in proliferation, apoptosis and invasion of colorectal cancer cells. Oncology letters.

[18] Aprelikova O, Yu X, Palla J, Wei BR, John S, Yi M, Stephens R, Simpson RM, Risinger JI, Jazaeri A, Niederhuber J (2010). The role of miR-31 and its target gene SATB2 in cancer-associated fibroblasts. Cell cycle (Georgetown, Tex).

[19] Mitra AK, Zillhardt M, Hua Y, Tiwari P, Murmann AE, Peter ME, Lengyel E (2012). MicroRNAs reprogram normal fibroblasts into cancer-associated fibroblasts in ovarian cancer. Cancer discovery.

[20] Fu LL, Wen X, Bao JK, Liu B (2012). MicroRNA-modulated autophagic signaling networks in cancer. The international journal of biochemistry & cell biology.

[21] Qased AB, Yi H, Liang N, Ma S, Qiao S, Liu X (2013). MicroRNA-18a upregulates autophagy and ataxia telangiectasia mutated gene expression in HCT116 colon cancer cells. Molecular medicine reports.

[22] Enkelmann A, Heinzelmann J, von Eggeling F, Walter M, Berndt A, Wunderlich H, Junker K (2011). Specific protein and miRNA patterns characterise tumour-associated fibroblasts in bladder cancer. Journal of cancer research and clinical oncology.

[23] Zhao L, Sun Y, Hou Y, Peng Q, Wang L, Luo H, Tang X, Zeng Z, Liu M (2012). MiRNA expression analysis of cancer-associated fibroblasts and normal fibroblasts in breast cancer. The international journal of biochemistry & cell biology.

[24] Nosho K, Igarashi H, Nojima M, Ito M, Maruyama R, Yoshii S, Naito T, Sukawa Y, Mikami M, Sumioka W, Yamamoto E, Kurokawa S, Adachi Y (2014). Association of microRNA-31 with BRAF mutation, colorectal cancer survival and serrated pathway. Carcinogenesis.

[25] Laurila EM, Kallioniemi A (2013). The diverse role of miR-31 in regulating cancer associated phenotypes. Genes, chromosomes & cancer.

[26] Wang S, Hu J, Zhang D, Li J, Fei Q, Sun Y (2014). Prognostic role of microRNA-31 in various cancers: a meta-analysis. Tumour biology.

[27] Gonzalez CD, Alvarez S, Ropolo A, Rosenzvit C, Bagnes MF, Vaccaro MI (2014). Autophagy, Warburg, and Warburg reverse effects in human cancer. BioMed research international.

